# Auraptene, a Major Compound of Supercritical Fluid Extract of Phalsak (*Citrus* Hassaku Hort ex Tanaka), Induces Apoptosis through the Suppression of mTOR Pathways in Human Gastric Cancer SNU-1 Cells

**DOI:** 10.1155/2015/402385

**Published:** 2015-08-13

**Authors:** Jeong Yong Moon, Hyeonji Kim, Somi Kim Cho

**Affiliations:** ^1^Subtropical Horticulture Research Institute, Jeju National University, Jeju 690-756, Republic of Korea; ^2^Faculty of Biotechnology, College of Applied Life Science, SARI, Jeju National University, Jeju 690-756, Republic of Korea

## Abstract

The supercritical extraction method is a widely used process to obtain volatile and nonvolatile compounds by avoiding thermal degradation and solvent residue in the extracts. In search of phytochemicals with potential therapeutic application in gastric cancer, the supercritical fluid extract (SFE) of phalsak (*Citrus* hassaku Hort ex Tanaka) fruits was analyzed by gas chromatography-mass spectrometry (GC-MS). Compositional analysis in comparison with the antiproliferative activities of peel and flesh suggested auraptene as the most prominent anticancer compound against gastric cancer cells. SNU-1 cells were the most susceptible to auraptene-induced toxicity among the tested gastric cancer cell lines. Auraptene induced the death of SNU-1 cells through apoptosis, as evidenced by the increased cell population in the sub-G1 phase, the appearance of fragmented nuclei, the proteolytic cleavage of caspase-3 and poly(ADP-ribose) polymerase (PARP) protein, and depolarization of the mitochondrial membrane. Interestingly, auraptene induces an increase in the phosphorylation of Akt, which is reminiscent of the effect of rapamycin, the mTOR inhibitor that triggers a negative feedback loop on Akt/mTOR pathway. Taken together, these findings provide valuable insights into the anticancer effects of the SFE of the phalsak peel by revealing that auraptene, the major compound of it, induced apoptosis in accompanied with the inhibition of mTOR in SNU-1 cells.

## 1. Introduction

Gastric cancer is a common malignant tumor, which is increasingly being reported. It remains at the second leading cause of cancer related death in the world for a long time [1]. Although infection with* Helicobacter pylori* is considered to be the primary cause, the cause of gastric cancer is multifactorial. There are several complicated reasons for the occurrence of gastric cancer such as food habit, lifestyle, and tobacco smoking [2]. Further, overweight and obesity are also correlated with increased risk of gastric cancer [3]. On the other hand, consumption of fruits or vegetables has been shown long since to decrease the risk of gastric cancer regardless of the anatomical location and the histological type [4].

The supercritical carbon dioxide is generally used as an extraction solvent since CO_2_ is inert and low toxic and requires short extraction time. Because of several such advantages, nowadays supercritical fluid extract (SFE) method is increasingly being used in the food, pharmaceutical, and environmental engineering sectors [5].* Citrus* has long been accepted as a rich source of anticancer compounds and several flavonoids have been reported to exhibit anticancer effect against various types of human cancer cells [6], especially the richest source of auraptene (7-geranyloxycoumarin) [7]. Auraptene has been known to have various pharmacological properties, such as cancer chemopreventive [8], antioxidant [9, 10], anti-inflammatory [11], antimicrobial, antigenotoxic, neuroprotective, and immunomodulatory properties in various animal models [7]. It has been reported to show apoptosis in human acute leukemia Jurkat T cells through ER stress mediated caspase-8 activation followed by stimulation of caspase cascade [12]. Furthermore, it has been reported as a chemopreventive agent against cancers of liver, skin, tongue, esophagus, and colon in rodent models while its effect in human cancer is not yet fully understood [13]. Moreover, little is known about the mechanism responsible for the apoptotic activity of auraptene against gastric cancer cells.

In the present study, the cytotoxicity of SFE of phalsak (*Citrus* hassaku Hort ex Tanaka) fruit in the human gastric cancer SNU-1 cells was assessed. We determined for the first time that auraptene, the major compound of phalsak peel SFE, induces cytotoxicity of SNU-1 cells with IC_50_ value ≤ 25 *μ*M. These results suggest that auraptene positively regulates apoptotic signaling by downregulating the mTOR pathway via feedback activation on Akt in SNU-1 cells.

## 2. Materials and Methods

### 2.1. Preparation of SFE

Dried peel (280.4 g) and flesh (371.1 g) were loaded separately into a 1 L thick-walled stainless steel thimble extraction cell and extracted at 50°C for 2 h. The extraction was performed using CO_2_ at 300 bar pressure in a diaphragm compressor (Haskell Co., Bellingham, WA, USA). The extracts (peel: 2.24 g, flesh: 1.75 g) were deposited in a separator attached to a metering valve and held in a circulating bath at 0°C. Finally, the extracts were collected into a clean vial and stored in aliquots at −20°C until analysis. The thawed sample was not frozen back for reuse.

### 2.2. Cell Culture

Human gastric cancer (AGS, MKN45, SNU-1, and SNU-16) cell lines were purchased from the Korean Cell Line Bank (KCLB, Seoul, Korea). Human embryonic kidney (HEK-293T) cells were kindly provided by Professor Jae Hoon Kim at the Faculty of Biotechnology, Jeju National University, Republic of Korea. Cells were maintained at 37°C in a humidified atmosphere under 5% CO_2_ in RPMI 1640 or DMEM containing 10% heat-inactivated FBS, 100 U/mL penicillin, and 100 *μ*g/mL streptomycin. Exponentially grown cells were treated with various concentrations of the solvent fractions, as indicated.

### 2.3. Cell Viability Assay

Cells were maintained in a humidified incubator at 37°C in a 5% CO_2_ atmosphere. Antiproliferative activity was determined by the cell viability assay. The effect of the samples on the viability of various cancer cell lines was determined by an MTT-based assay. Exponential-phase cells were collected and transferred to a 96-well microtiter plate (2 × 10^3^–5 × 10^4^ cells per mL) to detect cytotoxicity in the gastric cancer cell lines. The cells were incubated for 2 days with various concentrations of the fruit extract. The supercritical fluid extract was dissolved in DMSO and diluted in PBS to obtain a stock solution, which was stored at −20°C. After the incubation, 0.1 mg MTT (Sigma, St. Louis, MO, USA) was added to each well, and the cells were incubated at 37°C for 4 h. The medium was carefully removed. DMSO (150 *μ*L) was added to each well to dissolve the formazan crystals. The plates were read at 570 nm after the crystal had dissolved completely, using a Sunrise microplate reader (Sunrise, Tecan, Salzburg, Austria). The percent cell viability was calculated based on the following formula: mean value of (control group − treated group/control group) × 100%. All results were assessed in triplicate for each concentration.

### 2.4. Gas Chromatography-Mass Spectrometry (GC-MS) Analysis

GC-MS analysis was carried out using a Shimadzu GC-MS (Model QP-2010, Shimadzu Co., Kyoto, Japan) in the electron impact mode. The capillary column was an Rtx-5MS (30 m length, 0.25 mm internal diameter, and 0.25 *μ*M film thicknesses). Ionization was set at 70 eV and injector and detector were set at 250 and 290°C, respectively. The oven temperature was set at 60°C (isothermal for 2 min) and was ramped up to 250°C at 5°C/min (isothermal for 2 min) and up to 310°C at 8°C/min (isothermal for 5 min). Helium was used as the carrier gas at 1 mL/min with an injector volume of 1 *μ*L (1 : 10 split ratio). Supercritical fractions were dissolved in hexane (1 mg/mL), filtered through 0.2 *μ*m syringe filter (Advantec, Tokyo, Japan), and an aliquot of sample was injected into the GC-MS. The mass spectra of each compound were tentatively identified by comparing with Wiley 7th edited library data of the GC-MS system. The compound identity was further confirmed with the retention indices (RI) of standard compounds. RI values on the column were determined using a mixture of alkanes (C_7_–C_30_) which were run under identical conditions.

### 2.5. Microscopic Observation of Nuclear Morphology

Cells, placed in 6-well plates at 5 × 10^4^ cells/mL, were treated with the samples. After 24 h, 10 *μ*M of Hoechst 33342, a DNA-specific fluorescent dye, was added to the solution in each well and the plates were incubated for 10 min at 37°C. The stained cells were then observed under an Olympus fluorescence microscope.

### 2.6. Flow Cytometric Analysis

To determine cell cycle distribution analysis, 5 × 10^4^ cells/mL cells were plated in 6-well plate and treated with the samples for 24 h. After treatment, the cells were collected, fixed in 70% ethanol, washed in PBS (2 mM EDTA), resuspended in 1 mL PBS containing 1 mg/mL RNase and 50 mg/mL propidium iodide, incubated in the dark for 30 min at 37°C, and analyzed by FACS caliber flow cytometry (Becton Dickinson, USA). Data from 10,000 cells were collected for each data file.

### 2.7. JC-1 Analysis

Mitochondrial membrane potential (ΔΨm) was measured by flow cytometry using JC-1 staining. After treatment of the sample, the cells were incubated with 1 *μ*L of JC-1. After 10 min, cells were washed twice with PBS and immediately subjected to flow cytometric analysis. The percentage of cells in the high-red region or low-red and high-green region was measured under the different treatments.

### 2.8. Western Blot Analysis

After treatment, the cells were collected and washed twice with cold PBS. The cells were then lysed in lysis buffer (50 mM Tris-HCl, pH 7.5, 150 mM NaCl, 1% Nonidet P-40, 2 mM EDTA, 1 mM EGTA, 1 mM NaVO_3_, 10 mM NaF, 1 mM DTT, 1 mM PMSF, 25 *μ*g/mL aprotinin, and 25 *μ*g/mL leupeptin) and kept on ice for 30 min. The lysates were then centrifuged at 13,000 ×rpm at 4°C for 30 min; the supernatants were stored at −70°C until use. The protein concentration was determined by the bicinchoninic acid (BCA) protein assay kit (Pierce, Rockford, IL, USA). Aliquots of the lysates (60–100 *μ*g of protein) were separated by 7.5–15% SDS-PAGE and transferred onto a polyvinylidene difluoride (PVDF) membrane (Bio-RAD, HC, USA) using a glycine transfer buffer (192 mM glycine, 25 mM Tris-HCl, pH 8.8, and 20% methanol [v/v]). After blocking with 5% nonfat dried milk, the membrane was incubated for 2 h with primary antibodies followed by 30 min with secondary antibodies in milk containing Tris-buffered saline (TBS) and 0.1% Tween 20. All primary antibodies were used at a dilution of 1 : 1,000; HRP-conjugated goat anti-rabbit IgG (H+L) and HRP-conjugated goat anti-mouse IgG were used as secondary antibodies at a dilution of 1 : 5,000. The PVDF membrane was then exposed to X-ray film (AGFA, Mortsel, Belgium), and the protein bands were detected using a WEST-ZOL plus Western Blot Detection System (iNtRON, Gyeonggi-do, Korea).

### 2.9. Statistical Analysis

All results were expressed as the mean ± standard deviation. One-way analysis of variance using SPSS v 12.0 software package was applied. All assays were performed in triplicate. Values of ^*∗*^ *P* < 0.01 were considered to be statistically significant.

## 3. Results

### 3.1. Cytotoxic Effect and Chemical Composition of Supercritical Fluid Extracts

**Figure 1:**
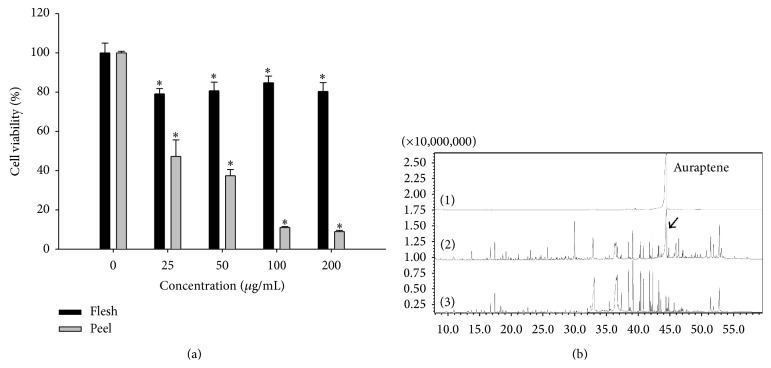
Cell viability on SNU-1 cells and GC-MS chromatogram of the SFEs of Jeju* Citrus* hassaku Hort ex Tanaka (phalsak). (a) Viability was determined on the basis of MTT reduction assay. (Data represent the means SD of at least four independent experiments. ^*∗*^ *P* < 0.01 indicate statistically significant differences versus control group.) (b) GC-MS chromatogram of SFEs. ((1) Auraptene standard peak, (2) SFE of phalsak peel, and (3) SFE of phalsak flesh; arrow indicates auraptene peak.)

MTT assay was carried out to investigate the cytotoxic effect of SFEs of* Citrus* hassaku Hort ex Tanaka (phalsak) peel and flesh (25–200 *μ*g/mL) against human gastric cancer SNU-1 cells. The cytotoxic effect of the peel fraction was significantly greater than the flesh fraction against the gastric cancer cells (Figure 1(a)). The GC-MS analysis of SFEs of phalsak revealed 49 compounds and 43 compounds in peel and flesh, respectively (Table 1). Auraptene was found to be the major compound in peel SFE, while almost no auraptene was detected in the flesh SFE (Figure 1(b)).

### 3.2. Cytotoxic Effect of Auraptene

**Figure 2:**
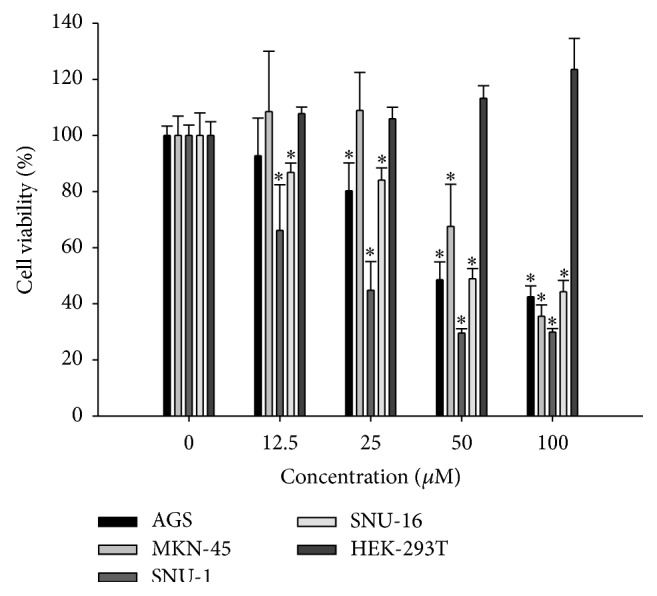
Cell growth inhibition by auraptene. Viability was determined on the basis of MTT reduction assay against various cancer cell lines and a noncancer cell line after treating with shown concentration of auraptene for 48 h. (Data represent the means SD of at least four independent experiments. ^*∗*^ *P* < 0.01 indicate statistically significant differences versus control group.)

Since the cytotoxic effect and the abundance of auraptene are significantly greater in the peel fraction than in the flesh fraction, we presume that auraptene is highly likely to be the most potent anticancer candidate present in the phalsak (*Citrus* hassaku Hort ex Tanaka) fruit. For this reason, we have studied the anticancer effects taking auraptene as a possible therapeutic candidate in the treatment of human gastric cancer. Although auraptene has previously been reported to have a potential of chemoprevention against various types of cancers such as liver, skin, tongue, esophagus, and colon in rodent models, its effect in human cancer is poorly reported [13]. This also substantiated our interest in anticancer effect of auraptene on human gastric cancer cells. As shown in Figure 2, auraptene reduced the viability of all types of tested gastric cancer cells in a dose-dependent manner. Among them, SNU-1 was the most sensitive with IC_50_ value ≤ 25 *μ*M. In contrast, auraptene did not pose cytotoxic effect against noncancer cell lines HEK-293T. Therefore, auraptene has high potential to treat gastric cancer cells without harming normal cells.

### 3.3. Effect of Auraptene on Cell Morphology, Cell Cycle Distribution, and ΔΨm

**Figure 3:**
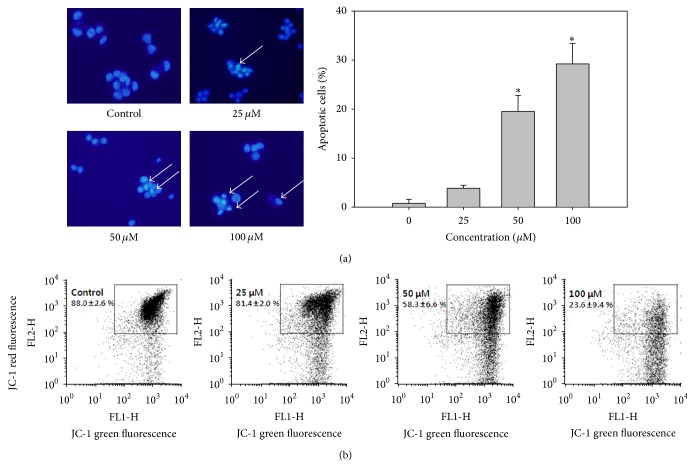
Nuclear Hoechst 33342 staining, cell cycle analysis, and determination of mitochondrial membrane potential. (a) Cells were treated with the indicated concentration of auraptene (25–100 *μ*M) for 24 h and then stained with Hoechst 33342. Stained cells were visualized under a fluorescence microscope. (b) Mitochondrial membrane potential was determined by FACS caliber after being treated with given doses of auraptene. (Data represent the means SD of at least four independent experiments. ^*∗*^ *P* < 0.01 indicate statistically significant differences versus control group.)

Morphological changes such as cell shrinkage, blebbing, condensation, and fragmentation of chromatin are associated with apoptotic cell death. To evaluate the effect of auraptene on proliferation and to characterize auraptene-induced apoptosis, SNU-1 cells were treated with or without various concentrations of auraptene and stained with Hoechst 33342 and then observed under fluorescence microscopy. As shown in Figure 3(a), after treatment with 25, 50, and 100 *μ*M auraptene, SNU-1 cells showed morphological changes, including condensed and fragmented chromatin with dose-dependently increased apoptotic bodies (arrows). Cell cycle regulation is the major regulatory mechanism of cell growth. Many cytotoxic agents arrest the cell cycle at the G1, S, or G2/M phases, cause cells to accumulate in the sub-G1 phase, and then induce apoptotic cell death. In this study, signs of apoptosis were indicated by the increased sub-G1 SNU-1 cells after auraptene treatment in a concentration dependent manner. The sub-G1 population was increased from 4.80% (0 *μ*M) to 23.45% (100 *μ*M). In addition, low concentration of auraptene (25 and 50 *μ*M) caused a significant increase in the G1 population (from 55.75% to 61.34 and 75.40%, resp.) (Table 2). These data suggest that auraptene inhibits SNU-1 cell proliferation by inducing G1 cell cycle arrest at relatively lower concentrations and causing the accumulation of sub-G1 cells at higher concentrations. As depicted in Figure 3(b) auraptene caused a decrease in the mitochondrial membrane potential (ΔΨm) in a concentration dependent manner, from 85.66% at control to 12.13% at 100 *μ*M. From these results it is clear that auraptene caused apoptotic cell death in SNU-1.

### 3.4. Effects of Auraptene on the Regulation of Apoptosis Related Proteins and PI3K/Akt/mTOR Signaling Pathway

**Figure 4:**
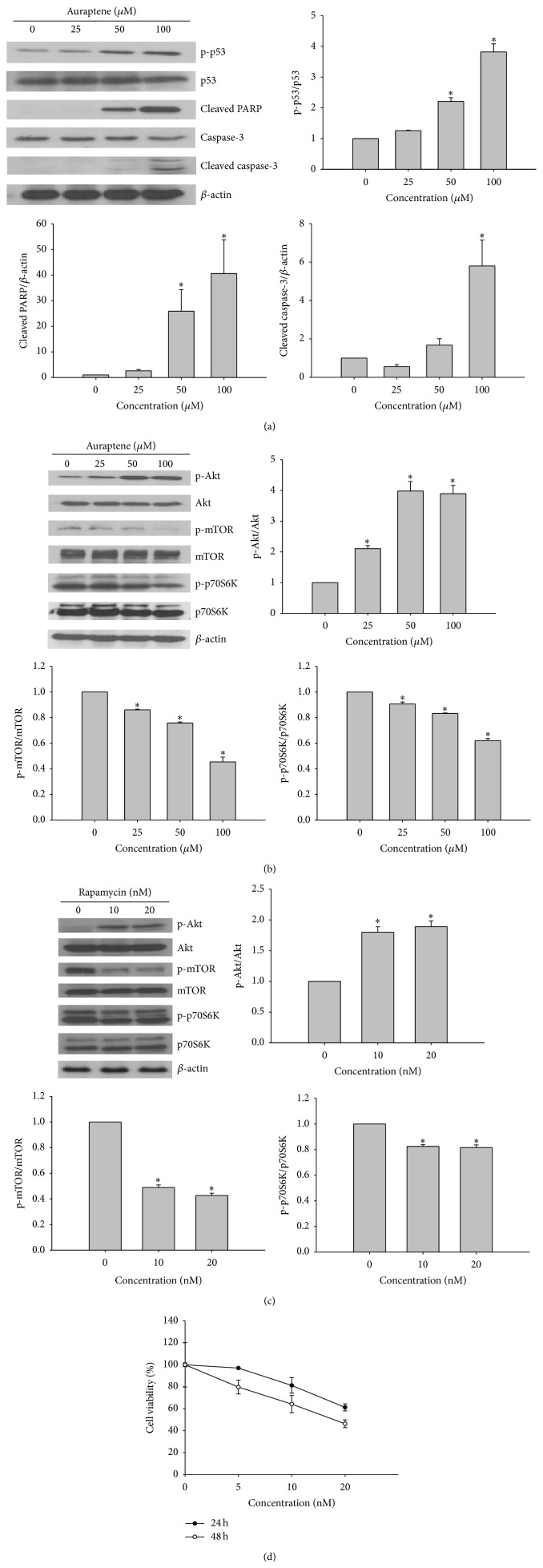
Western blot analysis of apoptosis and PI3K/Akt/mTOR related protein expression. (a) Cells were lysed after incubation with different concentrations of auraptene (25–100 *μ*M). After 24 h, cell lysates were subjected to western blotting as mentioned in the method. (b) Western blotting for the effect of auraptene on Akt/mTOR related proteins in SNU-1 cells. (c) Cells were treated with rapamycin for 24 h in SNU-1 cells. *β*-actin was used as an internal control. (d) Cell growth inhibition by rapamycin. Viability was determined on the basis of MTT reduction assay against SNU-1 cells for 24 and 48 h. (Data represent the means SD of at least four independent experiments. ^*∗*^ *P* < 0.01 indicate statistically significant differences versus control group.)

Apoptosis is regulated by various types of modulators, such as caspases [14]. To determine the mechanism of auraptene-induced apoptosis, the regulation of apoptotic proteins was examined by western blotting. The protein level of effector caspase-3 was decreased, whereas the level of cleaved form of caspase-3 was increased dose-dependently. Further, the level of proteolytically cleaved PARP was increased in a dose-dependent manner (Figure 4(a)). Cellular stress can activate p53, which induces cell cycle arrest or apoptosis. When DNA damage occurs, activated p53 halts the progression of cell cycle in the G1 phase to provide time for DNA repair. As illustrated in Figure 4(a), auraptene dose-dependently induced the phosphorylation of p53 and caused dose-dependent downregulation of cyclin D1, indicating that auraptene could induce cell cycle arrest in SNU-1 (Table 2). The PI3K/Akt/mTOR pathway is an intracellular signalling pathway important in cell growth and apoptosis. Therefore, we examined whether auraptene can regulate the PI3K/Akt/mTOR signaling pathway. As shown in Figure 4(b), the level of phospho-mTOR was decreased accompanied by downregulation of p-p70S6K and downstream of mTOR proteins. However, the level of phospho-Akt was increased, which is reminiscent of the effect of rapamycin, the mTOR inhibitor. Therefore, we tested whether auraptene can induce negative feedback loop on Akt/mTOR signaling, the same as rapamycin (mTOR inhibitor) does in SNU-1 cells. As shown in Figure 4(c), both auraptene and rapamycin downregulate phosphorylation of mTOR and its downstream p70S6K but feedback activation of Akt in a similar pattern (Figure 4(c)). Altogether, these results suggest that auraptene could induce p53 dependent cell cycle arrest and apoptosis and furthermore, it could suppress the proliferation of SNU-1 cells by downregulation of mTOR downstream signalling.

## 4. Discussion

The use of carbon dioxide in the supercritical state has been shown to be an effective method for obtaining bioactive molecules from plants. Recent studies reported that supercritical fluid extraction (SFE) is the process of separating one component from another using supercritical fluids as the extracting solvent. In this study, we determined the cytotoxic effect of SFE from peel and flesh of phalsak (*Citrus* hassaku Hort ex Tanaka) on human gastric cancer SNU-1 cells and demonstrated that phalsak peel SFE significantly inhibits the cell viability. In most of the studies on the volatile composition of* Citrus* fruits obtained by conventional methods of extractions, limonene was found in a proportion of > 94.8% [15, 16]. However, in the present study limonene was found to be extremely low levels in phalsak. The discrepancy can be attributed to the supercritical extracts as well as the geographical origin of the samples.

Apoptosis is a compactly regulated process controlled by several signaling pathways, such as the death receptors and mitochondrial pathways [17]. Mitochondria are known to play a central role in mediating “intrinsic death signals” and could therefore serve as a novel target for chemotherapy. Cell cycle regulation is the major regulatory mechanism of cell growth. Many cytotoxic agents arrest the cell cycle at the G1, S, or G2/M phases, cause cells to accumulate in the sub-G1 phase, and induce apoptotic cell death. Our results indicated that auraptene induces cell cycle arrest at low concentration and increases the population in sub-G1 phase in a dose-dependent manner (Table 2). In addition, cells were undergoing apoptosis by disturbed mitochondria membrane potential and activation of apoptosis related proteins. It has been suggested that chemically induced apoptosis is often associated with the loss of ΔΨm as a consequence of the leakiness of the inner mitochondrial membrane [18]. As depicted in Figure 3(b) auraptene caused a dramatic decrease in the mitochondrial membrane potential (ΔΨm) in a concentration dependent manner. The prevention of cancer is largely dependent on tumor suppressor protein p53. Cellular stress can activate p53 which induces cell cycle arrest or apoptosis. The choice between apoptosis or cell cycle arrest is influenced by many factors such as type of cell, stress, and action of p53 coactivators [19]. As illustrated in Figure 4(a) and Table 2, auraptene dose-dependently induced the phosphorylation of p53 and reduced the level of cyclin D1, indicating that activated p53 could halt the progression of cell cycle in G1 phase as well as induction of apoptosis.

The PI3K/Akt/mTOR pathway is involved in the regulation of cell growth and survival. Mammalian target of rapamycin (mTOR) is an essential component of intracellular signalling for cellular growth, mRNA translation, and metabolism. mTOR has been demonstrated to be involved in gene regulation and translation of specific transcripts when cells respond to environmental stimuli [20]. mTOR is regulated by mitogen-responsive pathways and it could be activated by PI3K/Akt pathways [21, 22]. However, previous studies suggest that inhibition of p70S6K by the mTOR inhibitor rapamycin results in an increase of the IGF-1R/IRS-1/PI3K (insulin-like growth factor receptor/insulin receptor substrate-1/phosphoinositide 3-kinase) signaling and activation of Akt. It has been reported that p70S6K mediates phosphorylation of IRS-1 inhibitory serine sites (S312 and/or S636/639) which lead to IRS-1 degradation [23, 24]. Thus, suppression of p70S6K activity by rapamycin may prevent inhibitory IRS-1 phosphorylation, thereby stabilizing IRS-1. An increase in IRS-1 adapter protein levels may induce Akt activity by augmenting IGF-1R signaling to PI3K/Akt. These data indicate that mTOR inhibition by rapamycin induces negative feedback activation of Akt that is IGF-1R/PI3K dependent. Interestingly, we noticed auraptene-induced phosphorylation of Akt but downregulation of mTOR and its downstream p70S6K (Figure 4(b)), indicating auraptene has effect of mTOR inhibition the same as rapamycin does in SNU-1 cells. Using human non-small cell lung cancer (NSCLC) cells, Sun et al. reported that mTOR inhibition by rapamycin induces activation of survival pathways involving increase of Akt and eIF4E phosphorylation. They showed that prevention or disruption of the activation of Akt and eIF4E enhanced rapamycin-mediated growth inhibition, indicating that the induced activation of Akt and eIF4E survival pathways counteracts the mTOR inhibitor's effect on the growth of human cancer cells [25]. It remains possible that mTOR inhibition induces Akt activation by other unknown mechanisms, such as indirect activation of mTOR-rictor by auraptene, which require further elucidation. Currently, the possibility that PI3K inhibitor and/or IGF-1R antibody in combination with auraptene can exhibit enhanced (synergistic) effects on the growth of human gastric cancer cells is under investigation in our laboratory.

Taken together, we found that auraptene, a major component in phalsak peel SFE, induces cell cycle arrest and apoptosis in SNU-1 cells via activation of p53 and inhibition of mTOR signaling pathway accompanied by negative feedback activation of Akt.

## Figures and Tables

**Table 1:** Identification of compounds present in supercritical fluid extract of phalsak fruit by GC-MS.

Name^a^	RT^b^	Peel^c^	Flesh^c^
*β*-myrcene	8.031	0.34	—
2-Octenal	9.764	—	0.49
Linalool	10.957	0.69	0.85
p-Menth-1-en-8-ol, (S)-(−)-	13.776	1.39	0.41
2,4-Dimethylbenzaldehyde	14.529	—	0.42
2-Ethylcyclohexanone	15.299	—	0.34
(E)-2-Decenal	15.777	—	0.21
*α*-Citral	16.057	0.22	—
(E,E)-2,4-Decadienal	16.727	1.43	0.98
Octadecyl vinyl ether	17.885	—	0.13
Citronellyl acetate	18.321	0.28	—
2,4-Dimethyl-5-oxo-heptanal	18.350	—	0.87
Undec-2-enal	18.654	—	0.28
Geranyl acetate	19.164	—	0.22
*β*-cubebene	19.517	0.39	—
Decylacetate	19.853	0.27	—
Limonen-10-yl acetate	19.999	0.18	—
trans-Caryophyllene	20.373	0.10	—
Perilla acetate	20.646	0.13	—
(E)-Geranylacetone	21.049	0.16	0.16
3,7-Dimethyloct-1-en-3,7-diol	21.146	0.52	—
Oleic acid	21.889	—	0.47
2,4-Di-tert-butylphenol	22.610	0.28	0.4
Sesquisabinene hydrate	22.857	0.16	—
*δ*-Cadinene	23.026	0.81	—
Dihydroactinidiolide	23.315	0.2	0.12
Lauric acid	23.822	0.38	0.33
Nerolidol	23.885	0.12	—
(−)-Caryophyllene oxide	24.645	0.40	0.17
Diisobutylamine	25.236	—	0.18
Spathulenol	25.681	—	0.21
*β*-sinensal	27.172	0.13	—
Duvatriendiol	27.916	0.20	—
Myristic acid	28.487	0.28	—
6-Tridecen-6-al	28.556	0.25	—
(−)-Loliolide	28.989	0.26	—
N-Butylbenzenesulfonamide	29.256	0.13	0.24
Nootkatone	29.93	5.83	0.15
Platambin	30.964	0.12	—
Leden alkohol	31.551	0.25	—
Farnesyl acetone A	31.922	0.31	—
Methyl palmitate	31.974	—	0.53
Heptadecene-(8)-carbonic acid-(1)	32.349	0.37	—
2-hydroxycyclopentadecan-1-one	32.433	—	1.79
Palmitic acid	32.830	4.56	—
Pentadecylic acid	33.024	—	10.77
Ethyl palmitate	33.360	—	0.11
Pentadecanoic acid, 14-bromo-	34.216	—	0.2
cis*-*Octadec-9-enal	34.360	—	0.29
6-Tridecen-6-al	34.730	0.36	—
Manool	34.985	—	0.37
n-Nonadecane	35.381	—	1.48
Methyl oleate	35.575	0.23	—
Methyl linoleate	36.248	3.61	—
Heptadecene-(8)-carbonic acid-(1)	36.403	3.46	—
Oleic acid	36.599	—	20.79
Methyldiethylborane	36.631	1.23	—
N-Hentetracontanol-1	37.985	—	0.45
n-Hexatriacontane	39.026	2.43	—
3-Methylheptadecane	40.266	1.53	—
n-Hexatriacontane	42.212	1.91	—
n-Tetracosane	43.068	—	0.69
Auraptene	44.453	20.77	—
2,2-Dimethyl-3-(3-methyl-5-phenylsulfanyl-pent-3-enyl)-oxirane	45.903	3.98	—
Lycopersen	46.323	1.97	0.18
trans-Caryophyllene	48.047	0.22	—
3-Adamantan-1-yl-2-amino-propionic acid	49.247	—	0.23
*α*-tocopheryl-*β*-d-mannoside	49.721	0.84	—
Vitamin E	49.755	—	0.21
4′,5,6,7,8-Pentamethoxyflavone	50.677	1.29	—
*δ*-5-Ergosterol	51.344	3.51	2.14
Stigmasterol	51.768	1.80	0.67
Obtusifoliol	52.290	0.43	0.3
24-*β*-Ethyl-5-*δ*-cholesten-3-*β*-ol	52.678	—	3.51
Clionasterol	52.714	6.34	—
Fucosterol	52.910	—	0.24
3,3′,4′,5,5′,7,8-Heptamethoxyflavone	53.036	2.33	—
9,19-Cyclolanost-24-en-3-ol, acetate	55.050	—	0.13
Friedelan-3-one	57.061	0.31	—

^a^Supercritical CO_2_ extract of fruit compounds tentatively identified based on retention index and elution order as well as the fragmentation pattern described in the literature.

^b^Retention time.

^c^Relative peak area percentage (peak area relative to the total peak area %).

**Table 2:** The percentage of SNU-1 in each phase after treatment with auraptene for 24 h.

	Auraptene (*μ*M)			
	0	25	50	100
Sub-G1	4.80 ± 1.36	7.34 ± 3.17	8.77 ± 4.45	23.45 ± 7.34
G1	55.75 ± 0.91	61.34 ± 2.19	75.40 ± 6.40	51.36 ± 4.77
S	14.37 ± 0.67	12.08 ± 1.35	6.54 ± 1.49	10.36 ± 0.13
G2/M	25.59 ± 0.54	19.72 ± 3.41	9.65 ± 2.10	15.38 ± 3.28
